# Ripretinib: First Approval

**DOI:** 10.1007/s40265-020-01348-2

**Published:** 2020-06-23

**Authors:** Sohita Dhillon

**Affiliations:** Springer Nature, Private Bag 65901, Mairangi Bay, Auckland, 0754 New Zealand

## Abstract

Ripretinib (QINLOCK™) is a novel type II tyrosine switch control inhibitor being developed by Deciphera Pharmaceuticals for the treatment of KIT proto-oncogene receptor tyrosine kinase (KIT)-driven and/or platelet derived growth factor receptor A (PDGFRA)-driven cancers, including gastrointestinal stromal tumour (GIST). Ripretinib inhibits KIT and PDGFRA kinase, including wild-type, primary and secondary mutations, as well as other kinases, such as PDGFRB, TIE2, VEGFR2 and BRAF. In May 2020, oral ripretinib received its first approval in the USA for the treatment of adult patients with advanced GIST who have received prior treatment with ≥ 3 kinase inhibitors, including imatinib. The US FDA, Health Canada and the Australian Therapeutic Goods Administration collaborated on the review of the ripretinib new drug application in this indication as part of Project Orbis; regulatory review in Australia and Canada is ongoing. Clinical development for GIST, solid tumours and systemic mastocytosis is underway in several countries worldwide. This article summarizes the milestones in the development of ripretinib leading to this first approval for the treatment of advanced GIST.

## Ripretinib (QINLOCK™): Key points

**Table:** 

A novel tyrosine switch control inhibitor being developed by Deciphera Pharmaceuticals for the treatment of KIT and PDGFRA-driven cancers.
Received its first approval on 15 May 2020 in the USA.
Approved for the treatment of adult patients with advanced GIST who have received prior treatment with ≥ 3 kinase inhibitors, including imatinib.

## Introduction

Gastrointestinal stromal tumour (GIST) is the most common mesenchymal tumour of the gastrointestinal tract, with a global annual incidence of 10–15 cases per million [1]. Approximately 80% of GIST have activating mutations in the *KIT* receptor tyrosine kinase gene and approximately 5–10% have activating mutations in the platelet-derived growth factor receptor alpha (*PDGFRA*) tyrosine kinase gene [2–4]. KIT and PDGFRA are structurally similar dual switch kinases, containing both an inhibitory switch and an activation loop switch that regulate kinase activity by binding to the kinase switch pocket [2, 4]. Oncogenic kinase mutations result in dysregulated switch control and constitutive activation of KIT and PDGFRA, leading to abnormal cell growth and survival [2, 4].

**Figure:**
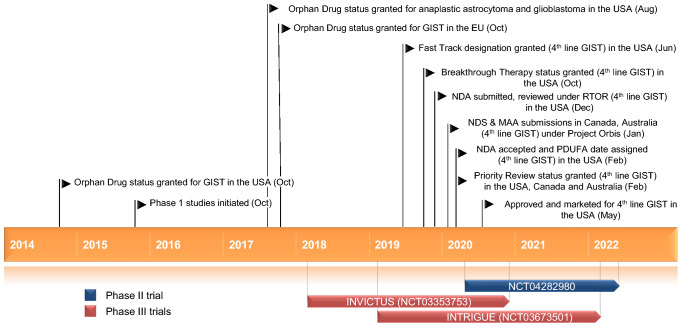
Key milestones in the development of ripretinib, focusing on its use in the treatment of gastrointestinal stromal tumour. *GIST* gastrointestinal stromal tumour, *NDA* New Drug Application, *NDS* New Drug Submission, *MAA* Marketing Authorisation Application, *PDUFA* Prescription Drug User Fee Act, *RTOR* Real-Time Oncology Review

Targeted therapy with tyrosine kinase inhibitors (TKIs) has revolutionized the treatment of GIST, with imatinib approved for patients with KIT positive unresectable and/or metastatic malignant GIST, sunitinib for those with imatinib-resistant GIST and regorafenib for patients with imatinib- and sunitinib-resistant GIST [3]. However, some patients have primary resistant GIST, and most patients with initial clinical benefit eventually develop resistance due to acquisition of secondary *KIT* mutations [2, 4]. These resistance mutations are quite heterogeneous, with multiple secondary mutations arising in individual patients [2, 4]. Given this heterogeneity, an unmet need existed for a drug that inhibited a broad spectrum of KIT and PDGFRA mutants, thus, blocking the various resistance mutations and limiting the impact of further resistance mutations [2, 4].

Ripretinib (QINLOCK™) is a novel type II, tyrosine switch control inhibitor designed to broadly inhibit activating and drug-resistant mutations in *KIT* and *PDGFRA*. It is being developed by Deciphera Pharmaceuticals for the treatment of KIT- and PDGFRA-driven cancers, including GIST, systemic mastocytosis and other solid tumours. On 15 May 2020, 3 months ahead of the PDUFA date [5], ripretinib received its first approval in the USA for the treatment of adult patients with advanced GIST who have received prior treatment with ≥ 3 kinase inhibitors, including imatinib [6]. The recommended dosage of ripretinib is 150 mg orally once daily with or without food until disease progression or unacceptable toxicity [6]. The US FDA, Health Canada and the Australian Therapeutic Goods Administration collaborated on the review of the ripretinib new drug application in this indication as part of Project Orbis; regulatory review in Australia and Canada is ongoing [5]. Clinical development for GIST, solid tumours and systemic mastocytosis is underway in several countries worldwide.

### Company Agreements

In June 2019, Deciphera Pharmaceuticals and Zai Lab entered into an exclusive license agreement to develop and commercialize ripretinib in Greater China (mainland China, Hong Kong, Macau and Taiwan) [7]. Under the terms of the agreement, Deciphera was to receive an upfront payment of US$20 million and was eligible to receive up to US$185 million in potential development and commercial milestone payments. Additionally, Deciphera was to receive royalties from low to high teens on annual net sales of ripretinib in Greater China. Zai Lab received exclusive regional development and commercialization rights for ripretinib in Greater China [7].

**Figure:**
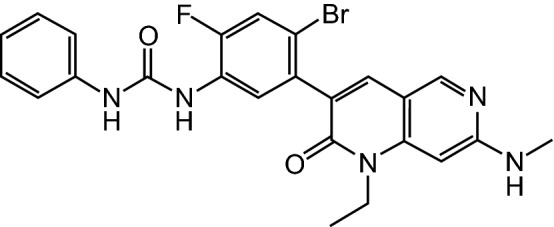
Chemical structure of ripretinib

## Scientific Summary

### Pharmacodynamics

Ripretinib is a type II “switch-control” kinase inhibitor that forces the activation loop (or activation “switch’’) into an inactive conformation [4]. This switch control mechanism has two components: (1) ripretinib is an antagonist, preventing switches from adopting a type I active state and (2) it is an agonist, stabilizing switches in the type II inactive state. Ripretinib and its active metabolite, DP-5439, potently inhibit the full spectrum of primary and secondary drug-resistant mutants of KIT and PDGFRA, including activation loop mutations previously thought to be targeted only by type I inhibitors. Ripretinib and DP-5439 exhibit comparable or superior cellular potency to that of the type I inhibitors midostaurin or avapritinib in inhibiting these activation loop mutants [4]. Ripretinib also inhibits other kinases in vitro, including PDGFRB, TIE2, VEGFR2, and BRAF [4, 6].

Ripretinib potently blocked proliferation and KIT phosphorylation and induced apoptosis in GIST cell lines derived from treatment-resistant patients, cell lines of other cancers with *KIT* or *PDGFRA* mutations (e.g., systemic mastocytosis and acute myeloid leukaemia) and cell lines transfected with *KIT*- or *PDGFRA*-activating mutations [4, 8]. Ripretinib synergized with the MEK inhibitors trametinib and binimetinib in inducing apoptosis in imatinib-sensitive and -resistant GIST and mastocytosis cell lines [9]. In addition, in vivo treatment with ripretinib and trametinib resulted in complete regression of tumour growth during treatment and long-term reduction in tumour growth after treatment in a GIST xenograft model [9].

Ripretinib at the recommended dosage of 150 mg once daily was not associated with a mean increase in the corrected QT interval of > 20 ms [6].

### Pharmacokinetics

The pharmacokinetic properties of ripretinib and its equally active metabolite, DP-5439, have been evaluated after single-dose administration in healthy subjects and multiple-dose administration in patients with advanced malignancies [6]. Following a single dose of ripretinib in patients with advanced malignancies, ripretinib area under the concentration–time curve from 0 to 24 h (AUC_24_) increased dose proportionally over a dose range of 20–250 mg, but ripretinib peak plasma concentration (C_max_) over this dose range and DP-5439 AUC_24_ and C_max_ over a dose range of 50–250 mg increased less than dose proportionally. After a single oral dose of ripretinib 150 mg, the median time to reach C_max_ was 4 h for ripretinib and 15.6 h for DP-5439. The time to steady state was 14 days for both ripretinib and DP-5439. The accumulation ratio AUC_12_ on day 15 of cycle 1 was 1.7 for ripretinib and 5.29 for DP-5439. There was no clinically significant difference in the exposure (C_max_ and AUC_24_) to ripretinib with a high fat meal and under fasted conditions; therefore, ripretinib can be taken without regard to food [6].

Ripretinib and DP-5439 are highly plasma protein bound to both human serum albumin (99.8% and 99.7%, respectively) and α-1 acid glycoprotein (99.4% and > 99.8%) [6]. Following a single oral dose of ripretinib 150 mg, the steady-state apparent volume of distribution of ripretinib was 307 L and that of DP-5439 was 507 L. Ripretinib and DP-5439 are metabolized mainly by CYP3A4; CYP2C8 and CYP2D6 play a minor role in the metabolism of ripretinib and CYP2C8, CYP2E1 and CYP2D6 play a minor role in the metabolism of DP-5439 [6]. After a single oral dose of ripretinib 150 mg, 34% of ripretinib and 6% of DP-5439 was excreted in the faeces and 0.02% of ripretinib and 0.1% of DP-5439 was excreted in the urine; the apparent clearance values of ripretinib and DP-5439 were 15.3 and 17.5 L/h and the elimination half-lives were 14.8 and 17.8 h, respectively [6].

Coadministration of ripretinib with a strong CYP3A inhibitor increased the exposure to ripretinib and DP-5439 (which may increase the risk of adverse reactions) and coadministration with a strong CYP3A inducer may decrease the exposure of ripretinib and DP-5439 (which may decrease ripretinib antitumour activity) [6].

**Table:** Features and properties of ripretinib

Class	Amines, antineoplastics, bromobenzenes, cyclopropanes, fluorinated hydrocarbons, naphthyridines, phenylurea compounds, pyrazoles, pyridines, small molecules
Mechanism of Action	Tyrosine kinase switch control inhibitor of a broad spectrum of activating and drug-resistant mutations in *KIT* and *PDGFRA*
Route of Administration	Oral
Pharmacodynamics	Forces the kinase activation loop into an inactive conformation
	Along with active metabolite (DP-5439) potently inhibits a broad spectrum of primary and secondary drug-resistant mutants of KIT and PDGFRA, including activation loop mutations
	Inhibits other kinases in vitro, including PDGFRB, TIE2, VEGFR2, and BRAF
	Potently blocked proliferation and KIT phosphorylation, and induced apoptosis in GIST cell lines derived from treatment-resistant patients and cell lines of other cancers with *KIT* or *PDGFRA* mutations
Pharmacokinetics	Median t_max_ 4 h for ripretinib and 15.6 h for DP-5439; time to steady state 14 days for both ripretinib and DP-5439
	Ripretinib and DP-5439 highly (> 99%) plasma protein bound to human serum albumin and α-1 acid glycoprotein
	Elimination half-life of ripretinib and DP-5439 14.8 and 17.8 h, respectively
Adverse events	
Any grade	Alopecia, fatigue, nausea, abdominal pain, constipation, myalgia, diarrhoea, decreased appetite, palmar-plantar erythrodysesthesia syndrome, vomiting
Grade 3 or 4	Abdominal pain, hypertension
Serious	Abdominal pain, anaemia, nausea, vomiting
ATC codes	
WHO ATC code	L01X-E (Protein kinase inhibitors)
EphMRA ATC code	L1H (Protein kinase inhibitor antineoplastics)
Chemical name	1-(4-bromo-5-[1-ethyl-7-(methylamino)-2oxo-1,2-dihydro-1,6-naphthyridin-3-yl]-2-fluorophenyl)-3-phenylurea

*t*_*max*_ time to reach peak plasma concentration

### Therapeutic Trials

#### INVICTUS Phase 3 Trial

Ripretinib significantly improved progression-free survival (PFS) relative to placebo in patients with advanced GIST who had received prior treatment with at least imatinib, sunitinib and regorafenib, according to results from the ongoing, randomized, double-blind, multinational, phase 3 INVICTUS trial (NCT03353753) [10]. Patients with unresectable, locally advanced or metastatic GIST were randomized 2:1 to ripretinib 150 mg once daily (intent-to-treat *n* = 85) or placebo (*n* = 44) until disease progression or unacceptable toxicity. Randomization was stratified according to prior lines of treatment (3 vs ≥ 4) and Eastern Cooperative Oncology Group (ECOG) performance status (0 vs 1 or 2). Following disease progression as assessed by Blinded Independent Central Review (BICR), patients were unblinded and those in the ripretinib group were permitted to increase the ripretinib dosage to 150 mg twice daily, continue ripretinib 150 mg once daily if there was clinical benefit or discontinue treatment; patients in the placebo group could switch to ripretinib 150 mg once daily or withdraw from the trial. Ripretinib significantly improved median PFS as assessed by BCIR relative to placebo (6.3 vs 1.0 months; primary endpoint), which corresponded to an 85% reduction in the risk of disease progression or death [hazard ratio (HR) 0.15; 95% CI 0.09–0.25; *p* < 0.0001]. The 6-month PFS rates with ripretinib and placebo were estimated to be 51% and 3.2%, respectively. The objective response rate (ORR) [assessed by BICR] in patients receiving ripretinib was 9.4% (partial responses in 8 of 85 patients) and in those receiving placebo was 0% (*p* = 0.0504); the median duration of response was not reached (data cut-off date of 31 May 2019). Median overall survival (OS) with ripretinib was 15.1 months compared with 6.6 months in placebo recipients (HR 0.36; 95% CI 0.21–0.62), including both the double-blind and open-label periods; the estimated 12-month OS rates in the respective groups were 65.4% and 25.9% [10].

**Table:** Key clinical trials of ripretinib

Drug(s)	Indication	Phase	Status	Location(s)	Identifier	Sponsor
Ripretinib, sunitinib	Advanced GIST	3	Recruiting	Multinational	NCT03673501; INTRIGUE; DCC-2618-03-002	Deciphera Pharmaceuticals
Ripretinib, placebo	Advanced GIST	3	Ongoing	Multinational	NCT03353753; INVICTUS; DCC-2618-03-001	Deciphera Pharmaceuticals
Ripretinib	Advanced GIST	EAP	Available	Multinational	NCT04148092; DCC-2618-99-001	Deciphera Pharmaceuticals
Ripretinib	Advanced GIST	2	Recruiting	China	NCT04282980; ZL-2307-002	Zai Lab Co., Ltd
Ripretinib	Advanced malignancies	1	Recruiting	Multinational	NCT02571036; DCC-2618-01-001	Deciphera Pharmaceuticals

*EAP* expanded access program, *GIST* gastrointestinal stromal tumour

#### Phase 1 Trial

An ongoing, open-label, multicentre, first-in-human, dose-escalation and -expansion phase 1 study (NCT02571036) demonstrated the clinical benefit of ripretinib in patients with advanced malignancies, including advanced GIST [11]. In the dose-escalation phase, patients received ripretinib 20–200 mg twice daily or 100, 150 or 250 mg once daily. The recommended phase 2 dosage of ripretinib was determined to be 150 mg once daily. This dosage was subsequently tested in 6 cohorts in the dose-expansion phase, including cohorts for patients with GIST based on prior lines of therapy (second-line, third-line, and at least fourth-line therapy; *n* = 31, 28 and 83, respectively). At the cut-off date of 10 August 2019, the ORRs with ripretinib in patients with GIST in second-, third- and at least fourth-line therapy were 19.4%, 14.3% and 7.2%, respectively. The median duration of PFS in the respective groups were 46.4 weeks (10.7 months), 36.3 weeks (8.3 months) and 23.9 weeks (5.5 months) and the median durations of response were 80 weeks (18.4 months), not estimable and 76.1 weeks (17.5 months) [11].

### Adverse Events

Ripretinib 150 mg once daily was generally well tolerated in patients with advanced GIST who had received prior treatment with at least imatinib, sunitinib and regorafenib, based on results from the phase 3 INVICTUS trial (NCT03353753) [6, 10]. The most common (incidence > 20%) any-grade adverse reactions with ripretinib were alopecia (52% in the ripretinib group vs 4.7% with placebo), fatigue (42% vs 23%), nausea (39% vs 12%), abdominal pain (36% vs 30%), constipation (34% vs 19%), myalgia (32% vs 12%), diarrhoea (28% vs 14%), decreased appetite (27% vs 21%), palmar-plantar erythrodysesthesia syndrome (21% vs 0%) and vomiting (21% vs 7%). The most common (incidence > 5%) grade 3 or 4 adverse reactions were abdominal pain (7% vs 4.7%) and hypertension (7% vs 0%) [6].

Serious adverse reactions occurred in 31% of patients receiving ripretinib and 44% of patients receiving placebo, with abdominal pain (4.7% vs 4.7%), anaemia (3.5% vs 2.3%), nausea (2.4% vs 0%) and vomiting (2.4% vs 0%) reported most frequently (incidence > 2%) with ripretinib [6, 12]. Treatment-related, treatment-emergent adverse events resulted in dose reductions in 6% of ripretinib and 2% of placebo recipients and treatment discontinuation in 5% and 2% of patients, respectively [10].

### Ongoing Clinical Trials

In addition to the ongoing phase 3 INVICTUS and phase 1 trials discussed in Sect. 2.2, the randomized, open-label, multinational, phase 3 INTRIGUE trial (NCT03673501) is recruiting patients to assess the efficacy of ripretinib versus that of sunitinib as second-line therapy in ≈ 426 patients with advanced GIST after prior treatment with imatinib [2]. The primary endpoint of the study is PFS as assessed by BICR and the key secondary endpoints are ORR (assessed by BICR) and OS [2]. Patients are also being recruited in an open-label, multicentre, phase 2 trial (NCT04282980) in China that will assess the efficacy, safety and pharmacokinetics of ripretinib in ≈ 35 patients with advanced GIST who have progressed on prior anticancer therapies. The primary endpoint of the study is PFS based on independent imaging review and secondary endpoints include ORR and OS. In addition, an Expanded Access Program (NCT04148092) is available outside the USA to provide access to ripretinib until the drug is approved and is commercially available in the patient’s country. Those eligible for the EAP are patients who have locally advanced unresectable or metastatic GIST who have received prior treatment with ≥ 2 US FDA-approved therapies and who do not meet the criteria to enrol in ongoing ripretinib studies [13].

## Current Status

On 15 May 2020 [5], ripretinib received its first approval in the USA for the treatment of adult patients with advanced GIST who have received prior treatment with ≥ 3 kinase inhibitors, including imatinib [6].
