# Correction to: Ripretinib: First Approval

**DOI:** 10.1007/s40265-020-01430-9

**Published:** 2020-11-06

**Authors:** Sohita Dhillon

**Affiliations:** Springer Nature, Private Bag 65901, Mairangi Bay, Auckland, 0754 New Zealand

## Correction to: Drugs (2020) 80:1133–1138 10.1007/s40265-020-01348-2

The article Ripretinib: First Approval, written by Sohita Dhillon, was originally published electronically in SpringerLink on 23 June 2020 without open access. After publication in volume 80, issue 11, pages 1133–1138 Deciphera Pharmaceuticals, LLC, requested that the article be Open Choice to make the article an open access publication. Post-publication open access was funded by Deciphera Pharmaceuticals, LLC. This article is licensed under a Creative Commons Attribution 4.0 International License, which permits use, sharing, adaptation, distribution and reproduction in any medium or format, as long as you give appropriate credit to the original author(s) and the source, provide a link to the Creative Commons licence, and indicate if changes were made. The images or other third party material in this article are included in the article’s Creative Commons licence, unless indicated otherwise in a credit line to the material. If material is not included in the article’s Creative Commons licence and your intended use is not permitted by statutory regulation or exceeds the permitted use, you will need to obtain permission directly from the copyright holder. To view a copy of this licence, visit http://creativecommons.org/licenses/by/4.0/.

The original article has been corrected.

